# Asthma and gender impact accumulation of T cell subtypes

**DOI:** 10.1186/1465-9921-11-103

**Published:** 2010-07-28

**Authors:** Matthew J Loza, Susan Foster, Eugene R Bleecker, Stephen P Peters, Raymond B Penn

**Affiliations:** 1 Department of Internal Medicine, Center for Human Genomics, Wake Forest University School of Medicine, Winston-Salem, NC, USA 27157

## Abstract

**Background:**

The "Th2 hypothesis for asthma" asserts that an increased ratio of Th2:Th1 cytokine production plays an important pathogenic role in asthma. Although widely embraced, the hypothesis has been challenged by various empirical observations and has been described as overly simplistic. We sought to establish whether CD3+CD28-mediated and antigen-independent accumulation of type 1 and type 2 T cells differs significantly between nonasthmatic and asthmatic populations.

**Methods:**

An ex vivo system was used to characterize the regulation of IFN-γ-producing (type 1) and IL-13-producing (type 2) T cell accumulation in response to CD3+CD28 and IL-2 stimulation by flow cytometry.

**Results:**

IL-13-producing T cells increased in greater numbers in response to antigen-independent stimulation in peripheral blood lymphocytes from female atopic asthmatic subjects compared with male asthmatics and both male and female atopic non-asthmatic subjects. IFN-γ^+ ^T cells increased in greater numbers in response to either antigen-independent or CD3+CD28-mediated stimulation in peripheral blood lymphocytes from atopic asthmatic subjects compared to non-asthmatic subjects, regardless of gender.

**Conclusions:**

We demonstrate that T cells from asthmatics are programmed for increased accumulation of both type 2 and type 1 T cells. Gender had a profound effect on the regulation of type 2 T cells, thus providing a mechanism for the higher frequency of adult asthma in females.

## Background

Asthma is frequently labeled a "Th2-like" disorder, based on an inflammatory profile in the asthmatic airway and is characterized by preferential elaboration of Th2 T cells and their cytokines [1-3]. After allergen challenge, elevated levels of the type 2 cytokines interleukin (IL)-4 and IL-13 are found in the airways of asthma subjects, associated with an influx of type 2 cells and eosinophils [1-3]. The preponderance of IL-4 and IL-13 relative to the type 1 cytokine IFN-γ is believed to promote feed-forward mechanisms of allergic inflammation in asthma. However, several observations suggest that the "Th2 hypothesis" as applied to asthma is overly simplistic. Although the ratio of type 2 cytokines to interferon (IFN)-γ is higher in asthmatics than in non-asthmatic subjects, levels of *both *cytokines are often dramatically increased in allergen-challenged asthmatics [4,5]. Respiratory infections, known to induce IFN-γ levels, have been proposed to protect against asthma development but also induce or worsen exacerbations in subjects with established asthma [1,4,6-8]. A number of studies also suggest that IFN-γ is important in the survival and activation of eosinophils [9,10].

Numerous factors regulate the accumulation of T cell subsets. Trafficking of T cells to sites of inflammation (e.g. to the airways in lungs), from peripheral blood and lymphoid tissues is one important regulatory component. Reduced numbers of regulatory T cells are hypothesized to influence allergen-specific increases in type 2 T cells in asthmatics [11]. The presence of regulatory stimuli and how T cell numbers increase in response to both antigenic and non-antigenic stimuli also determine absolute and relative numbers of type 1 and type 2 cells. Studies to date examining mixed T cell populations in vitro have characterized the effects of both antigenic, CD3-mediated and antigen-independent, bystander (e.g., IL-2, IL-15) stimuli, as well as "polarizing" effectors (IL-4, IL-12) on changes in T cell subset accumulation [12-14]. Whether T cells from asthmatics exhibit distinct regulatory features is not known.

To address this question we compared the regulation of T cell subtype accumulation in T cells obtained from both atopic asthmatic and non-asthmatic subjects. Our results reveal that under various conditions T cells from asthmatics appear programmed for increased accumulation of both type 2 and type 1 cells, and surprisingly, gender plays a role in the regulation of type 2 cells.

## Methods

### Subject populations

Peripheral venous blood was obtained from nonasthmatic (control) and atopic asthmatic human adults after informed consent was provided, in accordance with a Wake Forest University School of Medicine Institutional Review Board-approved protocol and the Helsinki Declaration. The criteria for being included in the atopic asthmatic population are provided in Additional File 1. All subjects refrained from taking asthma control medications at least 12 h prior to blood draw. Characteristics of asthmatic subjects studied in this work are presented in Table S1 in Additional File 1. Control subjects were healthy adults who had not been diagnosed or treated for asthma. All female control subjects, but <25% of male controls, underwent clinical testing to: 1) rule out asthma (history of symptoms, reversibility of FEV1 decrement, airway hyperresponsiveness as assessed by methacholine challenge, exclusion of other respiratory disorders); and 2) evaluate atopy by skin prick test to a panel of standard common aeroallergens. For some subjects, certain outcomes were not included in analyses because of missing data resulting from insufficient cell numbers.

### Cell culture

Peripheral blood lymphocytes (PBL), isolated by standard procedures [15] (>98% lymphocytes, as assessed by flow cytometry), were cultured in RPMI-1640 media supplemented with 5% pooled human plasma. Before culture (day 0), after 5-d culture with 50 U/ml recombinant human IL-2 + IL-12-neutralizing monoclonal antibody (αIL-12) + CD28 monoclonal antibody + CD3 monoclonal antibody (plate-bound), or after 6-d culture with 50 U/ml IL-2 + IL-12-neutralizing monoclonal antibody, cells were collected and assessed for viability and cell numbers with an automated cell counter (ViCell, BeckmanCoulter). Anti-IL-12 mAb was included to exclude any influence of IL-12 potentially released from the few contaminating monocytes (see Loza et al. [16]). For "T_H_2-polarized" cultures, 50 U/ml IL-2, 5 ng/ml IL-4 and IL-12-neutralizing monoclonal antibody were included in the CD3+CD28-stimulated cultures described above. For "T_H_1-polarized" cultures, 50 U/ml IL-2, 2 ng/ml IL-12, and TNF-α-neutralizing monoclonal antibody were included in the CD3+CD28-stimulated cultures described above (but without IL-12-neutralizing monoclonal antibody). See Additional File 1 for additional details about culture conditions.

### Cytokine production

After culture and counting an aliquot of cells, 2-4 × 10^6 ^cells/250 μl were stimulated for cytokine production (5-h with 2nM phorbol myristate acetate (PMA), 0.2 μg/ml calcimycin, 100 U/ml IL-2, 5 μM monensin). Intracellular IL-13 and IFN-γ accumulation by T cells was detected by immunofluorescence-flow cytometry as previously described [12,17]. Proportions of cytokine producing cells were analyzed within T cells, gated based on a standard viable forward and side scatter lymphocyte gate and staining for CD3/CD5. Analysis of IL-4, IL-5, and IL-10 was not included in the study because the proportion of positive-expressing cells in freshly PBL was too low (0.4%) to accurately assess in the majority subjects, including asthmatics (not shown).

### Analysis of proliferation and progenitor numbers

The number of divisions that IL-13^+ ^and IFN-γ^+ ^T cell progenitors undergo during culture was determined by analysis of carboxyfluorescein diactetate succinimydl ester (CFSE) dilution in CFSE-labeled PBL from female control and female asthmatic subjects, as previously described (12). Average division numbers and minimum progenitor numbers were calculated for experiments of CFSE-labeled PBL as described previously [12,15]. See Additional File 1 for details of CFSE analyses.

### Statistical analyses

Distributions of non-normalized data sets were significantly non-Gaussian. Therefore, significance of differences in medians between non-normalized data sets was tested with Mann-Whitney tests (two-tailed). Analyses of accumulation of cytokine^+ ^T cell subsets in culture were performed on data normalized as a percent of the respective day 0 values (day 0 = 100%) because of the variability in the absolute proportions of day 0 cytokine^+ ^T cells. Normalized data were log-transformed to obtain approximate normality of distribution. Significance of differences in transformed (geometric) means was tested by Student's t-tests (two-tailed, corrected for unequal variances). Significance of gender and asthma status on accumulation was tested by 2-way General Linear Model analyses, with up to 2-way interaction model and planned Tukey-Kramer multiple comparison tests. Statistical analyses were performed using GraphPad Prism (4.0.3; San Diego California USA, http://www.graphpad.com) and Number Cruncher Statistical Systems (NCSS, 2004; J. Hintze, Kaysville, Utah, http://www.NCSS.com) software.

## Results

### Type 1 and type 2 T cell subsets in freshly isolated PBL

In freshly isolated human PBL, distinct subsets of T cells are capable of producing IL-13 (IL-13^+^) and IFN-γ (IFN-γ^+^). The median proportions of IL-13^+ ^and IFN-γ^+ ^T cells did not differ significantly between non-asthmatic (control) and atopic asthmatic (asthmatic) subjects (IL-13^+^: 0.8%, interquartile ranges (IQR): 0.5 - 2.1%, *n *= 21; and 0.9%, IQR: 0.5 - 2.5%, n = 25, respectively; *p *= 0.56); IFN-γ^+^: (8.4%, IQR: 2.2 - 13.5, *n *= 20; and 5.4%, IQR: 2.7 - 14.0, *n *= 25, respectively; *p *= 0.71).

### Accumulation of type 1 and type 2 T cell subsets

#### CD3+CD28-mediated stimulation

To test whether T cell subsets in freshly isolated PBL differ in their response to antigenic stimulation, PBL were stimulated with CD3 and CD28 monoclonal antibody to mimic efficient antigenic stimulation by professional antigen-presenting cells (e.g., activated macrophages, dendritic cells). The median proportion of IL-13^+ ^T cells after 5-day culture with CD3+CD28-mediated stimulation did not significantly differ between control subjects and asthmatics (Table 1). In contrast, the median proportion of IFN-γ^+ ^T cells after 5-day culture was significantly higher in asthmatics than in control subjects.

**Table 1:** Proportions of IL-13^+ ^and IFN-γ^+ ^T cells after culture.

Stimulus *	Subset	Subjects	**Median **^**†**^	**IQR **^**†**^	*n*	***p ***^**‡**^
CD3+CD28	IL-13^+^	Control	0.8%	0.6 - 4.3	17	0.67
+IL-2		Asthma	1.8%	0.5 - 4.2	18	
						
	IFN-γ^+^	Control	2.9%	0.6 - 7.8	11	0.035
		Asthma	7.4%	5.1 - 20.2	19	
						
IL-2	IL-13^+^	Control	1.0%	0.7 - 3.2	24	0.0004
		Asthma	4.5%	2.5 - 5.3	18	
						
	IFN-γ^+^	Control	4.1%	2.4 - 10.3	19	0.0076
		Asthma	10.7%	7.2 - 24.2	19	

* After 5-day (CD3+CD28+IL-2) or 6-day (IL-2) culture of PBL (anti-IL-12 monoclonal antibody included in all cultures), proportions of cytokine-producing T cells were determined by flow cytometry.

^† ^Median and 25%-75% interquartile range (IQR) of the proportion of IL-13+ or IFN-γ^+ ^T cell subsets.

^‡ ^*p*-value for the significance of difference between control and asthmatic subjects (two-tailed Mann-Whitney test).

**Figure 1:**
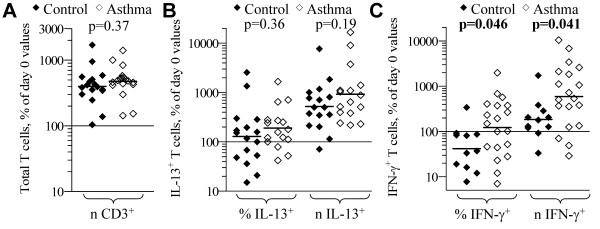
**CD3-stimulated accumulation of T cell subsets from control and asthmatic subjects**. PBL from control (filled symbols) and atopic asthmatic (open symbols) subjects were cultured 5-d with CD3+CD28 monoclonal antibody + IL-2 + anti-IL-12, and then stimulated and analyzed for cytokine production by immunofluorescence-flow cytometry. Each symbol indicates the proportion (%) and number (*n*) of: (A) total; (B) IL-13^+^; and (C) IFN-γ^+ ^T cells, expressed a percent of the respective day 0 values for each subject tested. Bars = geometric mean for each parameter. *p*-values from two-tailed student t-tests for differences in geometric mean between control and asthmatic subjects are shown.

After 5-day culture the total number of T cells from both asthmatic and control subjects increased ~400% above the day 0 number (Figure 1A). The number of IL-13^+ ^T cells from both asthmatic and control subjects likewise increased relative to the day 0 numbers, although there was not a significant mean increase in the proportion of IL-13^+ ^T cells from either group (Figure 1B). There was a significant impact of gender on the results (p = 0.0001), but no interaction between gender and asthma observed (results of stratified analyses are discussed below in "Gender effects on T cell subset accumulation" section). The proportion of IFN-γ^+ ^T cells decreased during culture for control subjects, but increased for asthmatic subjects, such that changes in proportion were different (*p *= 0.046) between groups (Figure 1C). There was a significant mean increase in number of IFN-γ^+ ^T cells during culture for both asthmatic and control subjects, and this increase was greater for asthmatics compared to control subjects (*p *= 0.041). Using starting (day 0) proportions of IL-13^+ ^and IFN-γ^+ ^as covariates in Analyses of Covariance (ANCOVA) did not change the significance of the above findings.

#### IL-4, IL-12 polarization effects

**Figure 2:**
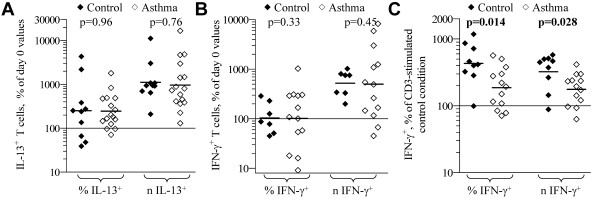
**Effects of 'classical' polarization conditions on accumulation of T cell subsets**. PBL from control and asthmatic subjects were cultured 5-d with CD3+CD28 monoclonal antibody + IL-2 and IL-4 + anti-IL-12 (**A**), IL-12 (B, C), or anti-IL-12 (**C**), and then stimulated and analyzed for cytokine production. Each symbol indicates the proportion (%) and number (*n*) of IL-13^+ ^(A) and IFN-γ^+ ^(B) T cells, expressed as a percent of respective the day 0 values for each subject tested. (C) Indicated are % and *n *of IFN-γ^+ ^T cells in the IL-12 condition, expressed as a percent of respective anti-IL-12 (control) condition values. Bars = geometric mean for each parameter. *p*-values from student t-tests for differences in geometric mean between control and asthmatic subjects are shown.

Under the 'classical Th2-polarization' culture condition of CD3+CD28-mediated stimulation plus IL-2, IL-4, and anti-IL-12, IL-13^+ ^T cells increased significantly in number, with no significant difference between control and asthmatic subjects (Figure 2A). Under the 'classical Th1-polarization' culture condition of CD3+CD28-mediated stimulation plus IL-2 and IL-12, IFN-γ^+ ^T cells from control subjects increased in number with a mean zero-change in proportion (Figure 2B), in contrast to that observed in the CD3-stimulated condition in the absence of IL-12 (Figure 1C). The mean change in CD3+CD28+IL-12-stimulated accumulation of IFN-γ^+ ^T cells was similar for control and asthmatic subjects. Because IFN-γ^+ ^T cells from asthmatics accumulate to greater numbers in CD3+CD28-stimulated cultures with IL-2 in the absence of IL-12, the response of T cells specifically to IL-12 was assessed by comparing IFN-γ^+ ^T cell accumulation in cultures with IL-2 + IL-12 relative to cultures with IL-2 + anti-IL-12 (Figure 2C). Relative to the IL-2 + anti-IL-12 condition, IFN-γ^+ ^T cell accumulation increased more in cultures with IL-12, with this increase in both number and proportion being significantly greater in control subjects than in asthmatics.

#### IL-2 stimulated accumulation

**Figure 3:**
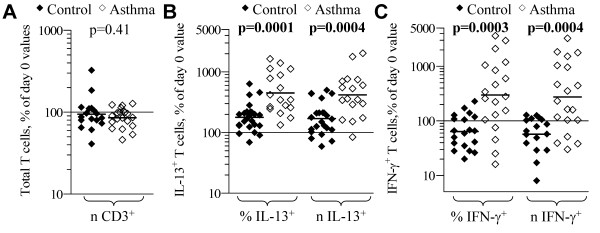
**IL-2-stimulated accumulation of T cell subsets**. PBL from control (filled symbols) and atopic asthmatic (open symbols) subjects were cultured 6-d with IL-2 + anti-IL-12, and then cells were stimulated and analyzed for cytokine production. Each symbol indicates the proportion (%) and number (*n*) of: (A) total; (B) IL-13^+^; and (c) IFN-γ^+ ^T cells, expressed as a percent of the respective day 0 values, for each subject. Bars = geometric mean for each parameter. *p*-values from two-tailed student t-tests for differences in geometric mean between control and asthmatic subjects are shown.

IL-13^+ ^T cells also proliferate in response to IL-2, independent of CD3/antigen-mediated stimulation, preferentially accumulating in cultures relative to the major IL-13^- ^T cell population (12, 15). Total T cell numbers did not significantly change in PBL from both control and asthmatic subjects in cultures with IL-2 (Figure 3A). In PBL stimulated with IL-2, the median proportion of IL-13^+ ^and IFN-γ^+ ^T cells after 6-day culture was significantly higher in asthmatics than in control subjects (Table 1). IL-13^+ ^T cells from both asthmatic and control subjects significantly increased relative to starting day 0 values, and this increase in accumulation was significantly greater in asthmatic than in control subjects (*p *≤ 0.0004) (Figure 3b). There was also a significant interaction (p = 0.003) between gender and asthma status for IL-2-stimulated increases in IL-13+ T cells, suggesting gender-specific differences for the observed associations with asthma (see "Gender effects on T cell subset accumulation" section below for discussion of stratified analyses). In control subjects, IFN-γ^+ ^T cells decreased in proportion (*p *= 0.01) and number (*p *= 0.008) in cultures with IL-2 (Figure 3C). In stark contrast, IFN-γ^+ ^T cells from asthmatic subjects increased in both proportion (*p *= 0.007) and number (*p *= 0.008), and the differences in accumulation were significantly different (*p *≤ 0.0004) between the control and asthma groups. For control subjects, IFN-γ^+ ^cells increased in proportion and number only when IL-12 was included in the culture (not shown). A role for IL-12 or IL-23 in the increase in IFN-γ^+ ^in PBL from asthmatics was excluded because IL-12p40-neutralizing monoclonal antibody were included in all cultures. Using starting (day 0) proportions of IL-13^+ ^and IFN-γ^+ ^as covariates in ANCOVA did not change the significance of the above findings.

### Gender effects on T cell subset accumulation

Race and gender may contribute to asthma risk, with asthma being more common in African Americans than in Caucasians (National Center for Health Statistics. Asthma: http://www.cdc.gov/nchs/fastats/asthma.htm.*CDC*. 2006), and, in adult asthmatics, more common in pre-menopausal females than in males (reviewed by Balzano et al. [18]). The asthma population used in this study consisted of 60% Caucasian and 40% African-Americans, and 55% females and 45% males. All data were analyzed to consider the cofactors of race and gender. There was no difference between Caucasian and African American asthmatics in accumulation (IL-2- and CD3- stimulated) of IL-13^+ ^or IFN-γ^+ ^T cells, and both Caucasian and African American asthmatic groups showed greater accumulation of IL-2-stimulated accumulation of IL-13^+ ^and IFN-γ^+ ^T cells compared to Caucasian controls (not shown). The number of African American control subjects was not sufficient to enable comparisons between racial groups in control subjects.

**Figure 4:**
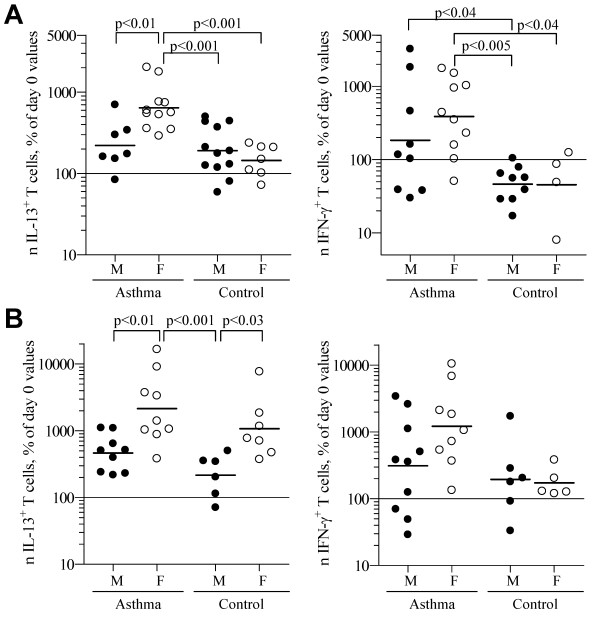
**Effects of gender and asthma on T cell subset accumulation**. Data obtained in the experiments for Figures 2 and 4 were reanalyzed, including both asthma and gender as independent variables in 2-way General Linear Model analyses of accumulation of IL-13^+ ^and IFN-γ^+ ^T cells. Each symbol indicates the number of: IL-13^+ ^(left panels) and IFN-γ^+ ^(right panels) T cells, expressed as a percent of the respective day 0 values for each subject after (A) 6-d culture with IL-2 + anti-IL-12 or (B) 5-d culture with CD3+CD28monoclonal antibody + IL-2 + anti-IL-12. Bars = geometric mean for each parameter. *p*-values from planned Tukey-Kramer multiple comparison tests are shown.

The data for IL-2-stimulated accumulation of IL-13^+ ^and IFN-γ^+ ^T cell accumulation were stratified to demonstrate the contributions of gender and asthma on accumulation (Figure 4). There was a significant interaction between asthma and gender for IL-2-stimulated accumulation of IL-13^+ ^T cells (*p *= 0.003) but not IFN-γ^+ ^T cells (*p *= 0.42). Female asthmatics had significantly greater IL-2-stimulated accumulation of IL-13^+ ^T cells than both male and female controls (*p *< 0.001 for both) and also male asthmatics (*p *< 0.01). For the 6 experiments in which CD8 staining was available, IL-2-stimulated accumulation of IL-13^+ ^T cells was greater in female vs. males asthmatics for both CD8^- ^(mostly CD4^+^) and CD8^+ ^T cell subsets (Figure S1 in Additional File 1). IFN-γ^+ ^T cell accumulation was increased in both CD8^+ ^and CD8^- ^T cell subsets in asthmatics regardless of gender (data not shown).

All but one of the female control subjects were atopic, as determined by skin prick tests to a panel of common aeroallergens. The differences between female asthmatics and female controls remained significant (p < 0.001) when excluding the one nonatopic female control subject (not shown). Skin prick tests were performed on only 6 of the male control subjects, all of whom tested positive. The mean increase in IL-13^+ ^T cells remained similar for atopic nonasthmatic males compared to atopic nonasthmatic females, as observed for the main analysis not restricted by atopy status (not shown). These results demonstrate that the observed associations for asthma- and gender-specific increases in IL-13^+ ^T cells is a function of atopic asthma per se, rather than a function of atopy status alone.

The difference in IL-2-stimulated accumulation of IL-13^+ ^and IFN-γ^+ ^T cells between female and male asthmatics remained significant when data from lung function studies (e.g., methacholine PC20, percent predicted FEV_1_, % reversal in FEV_1 _by albuterol, percent predicted forced vital capacity (FVC), FVC/FEV_1_) and other factors (age, serum IgE, body mass index) were considered as covariates in *GLM *analyses (not shown). These covariates were not included in the final *GLM *models because none of these variables significantly differed between male and female asthmatics, nor correlated with extent of T cell subset accumulation (see Table S1 in Additional File 1). For CD3+CD28-stimulated accumulation of IL-13^+ ^T cells, there was a significant difference between male and female subjects overall (*p *= 0.0001) and within asthmatic (*p *< 0.01) and control (*p *< 0.03) populations, but not between asthma and control populations within the same gender. An influence of gender on CD3+CD28-stimulated accumulation of IFN-γ^+ ^T cells was not detected.

### Lack of influence of inhaled corticosteroids

Although inhaled corticosteroids for asthma control was withheld for at least 12-h before blood draw, it is still possible that this anti-inflammatory drug may impact the peripheral blood T cells. To control for this potential confounder, two-way ANOVA were performed to adjust for regular inhaled corticosteroid use and gender for T cell subset accumulation in the asthma population (Table 2). Regular steroid use did not impact IL-2-stimulated accumulation of IL-13^+ ^and IFN-γ^+ ^T cells. Regular steroid use tended to decrease CD3+CD28-stimulated accumulation of IL-13^+ ^and IFN-γ^+ ^T cells, regardless of gender (no interaction effect). This observation actually strengthens the observed increased accumulation of IFN-γ^+ ^T cells in asthmatics compared to controls, as regular steroid use would tend to lower the mean accumulation of IFN-γ^+ ^T cells in the asthma population.

**Table 2:** Impact of regular inhaled corticosteroid use on T cell subset accumulation.

Culture stimulus	T cell subset	Asthma group		Accumulation, % of day 0 numbers *			**p-value **^**†**^		
				
		Gender	**Steroid **^**•**^	Geo. Mean	Geo. Mean ± S.D.	n	Gender	Steroid	Interaction
IL-2	IL-13^+^	Female	No	622	326 - 1184	6	0.0016	0.16	0.22
			Yes	662	342 - 1280	5			
		Male	No	116	73 - 184	2			
			Yes	285	155 - 525	5			
							
	IFN-γ^+^	Female	No	677	229 - 1994	6	0.41	0.28	0.40
			Yes	170	69 - 420	4			
		Male	No	206	101 - 423	3			
			Yes	173	21 - 1415	6			
							
CD3+CD28	IL-13^+^	Female	No	5427	1281-22,996	3	0.0016	0.044	0.33
+ IL-2			Yes	1352	571 - 3,201	6			
		Male	No	656	393 - 1,094	3			
			Yes	388	204 - 738	6			
							
	IFN-γ^+^	Female	No	3730	886 - 15,702	3	0.073	0.023	0.98
			Yes	691	241 - 1,982	6			
		Male	No	1035	396 - 2,707	3			
			Yes	186	36 - 962	7			

* Two-way General Linear Model analysis was performed to adjust for the impact of regular steroid use and gender on accumulation of T cell subsets from asthmatic subjects. Geo. Mean = geometric mean for the numbers of IL-13+ or IFN-γ+ T cells relative to the respective day 0 numbers after culture with the indicated culture stimulus. S.D. = standard deviation.

^† ^The significance of differences in geometric mean between males and females (gender) and between asthma subjects that use or don't regularly use inhaled steroids (steroid), and the significance of interaction between gender and steroid effects.

^• ^Indicates whether asthma subjects regularly use inhaled corticosteroids for asthma control. Note that all asthma subjects refrained from using asthma control medications 12-h before blood draw. No subjects regularly used oral corticosteroids for asthma control.

To confirm that asthma status, and not differences in steroid use, determines the increased accumulation of IL-13^+ ^and IFN-γ^+ ^T cells, female asthmatics and controls were compared, restricting the analysis to those subjects that do not use inhaled steroids. The significance levels for differences between female asthmatics and controls for IL-2-stimulated accumulation of IL-13^+ ^and IFN-γ^+ ^T cells and CD3+CD28-stimulated accumulation of IL-13^+ ^and IFN-γ^+ ^T cells were p = 0.0003, 0.08, 0.006, and 0.004 respectively (data not shown). Overall these data suggest that regular steroid use before the 12-h withdrawal of asthma-control medications do not impact the significance of the findings in this study.

### Mechanisms mediating differences in accumulation

**Figure 5:**
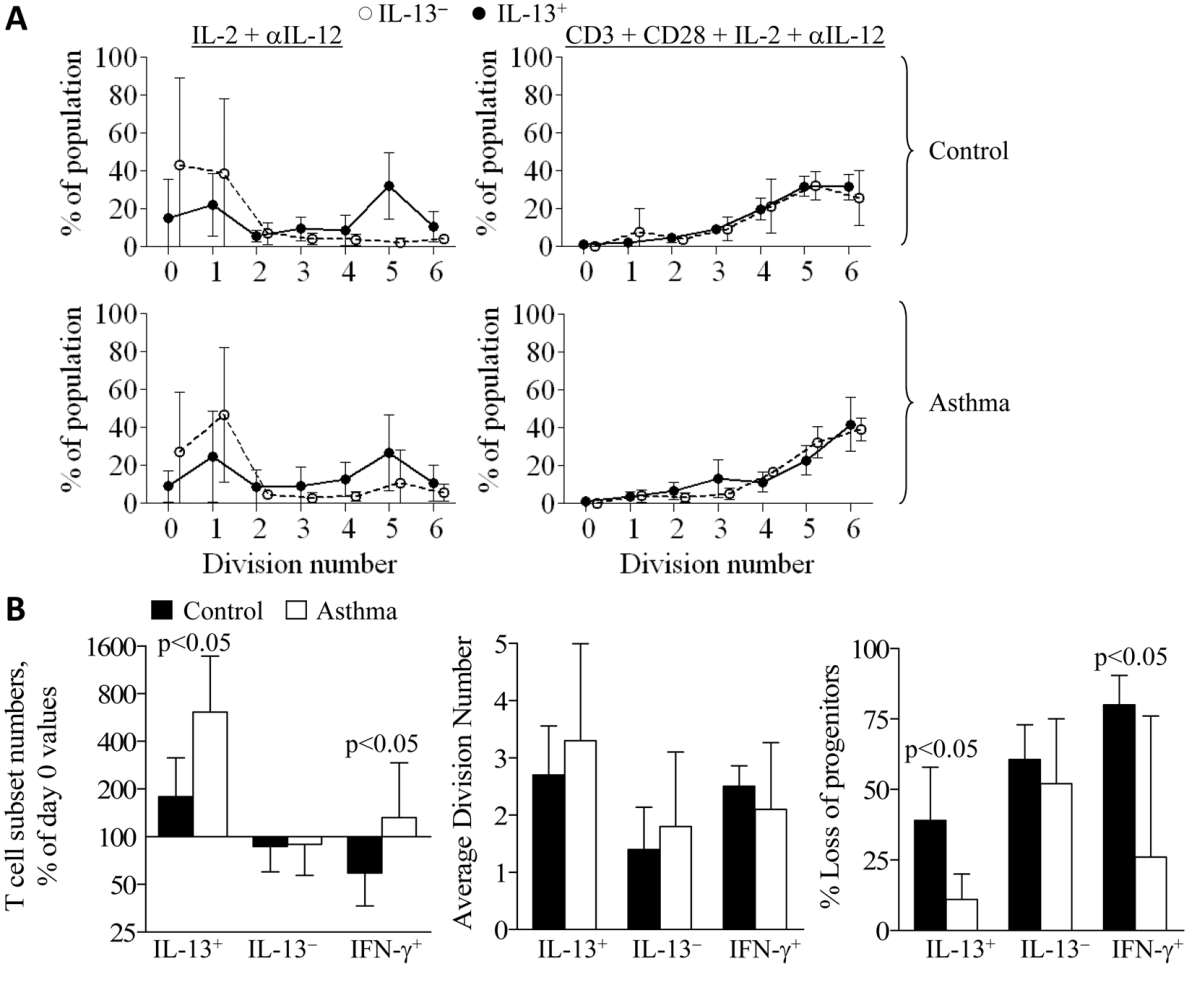
**Proliferation of IL-13^+ ^T cells from control and asthmatic subjects**. CFSE-labeled PBL from female control (*n *= 6) and female atopic asthmatic subjects (*n *= 7) were cultured 6-d with IL-2 and anti-IL-12 or 5-d with CD3+CD28 monoclonal antibody + IL-2 + anti-IL-12, and then stimulated and analyzed for cytokine production. (A) Proportions (mean ± S.D.) of cells in IL-13^+ ^and IL-13^- ^T cell populations (y-axis) that had undergone the indicated number of divisions (x-axis). (B) Compiled analysis for: cell numbers of the indicated T cell population, expressed as a percent of day 0 values, geometric mean + 95% CI (left panel); the average number of divisions the indicated T cell population underwent during culture, mean ± SD (middle panel); the percentage loss of progenitors of the indicated T cell population, mean ± SD (right panel). * *p *< 0.05 for difference in (geometric) mean.

Previous studies demonstrated that IL-2 stimulates accumulation of IL-13^+ ^T cells by preferentially inducing proliferation of IL-13^+ ^T cells [12,15]. A potential mechanism to explain the greater accumulation of IL-13^+ ^in female asthmatics is a difference in IL-2-stimulated proliferation. Representative plots of division numbers (determined by CFSE fluorescence peaks) and the proportion of IL-13^+ ^or IL-13^- ^T cells that had undergone that number of divisions are shown in Figure 5A. In IL-2-stimulated cultures, most IL-13^+ ^T cells from female control and asthmatic subjects underwent approximately 4-5 divisions in 6 days, whereas few IL-13^- ^T cells divided at all. There was no significant difference between female control and asthmatic subjects for the average number of divisions by IL-13^+ ^T cells (Figure 5B). In CD3+CD28-stimulated cultures, IL-13^+ ^and IL-13^- ^T cells proliferated to a similar extent, undergoing ~4-6 divisions in 5 days, with no significant differences between control and asthmatic subjects (not shown).

The theoretical maximum increase in IL-13^+ ^T cells expected during culture, on the assumption that no IL-13^+ ^T cells are lost (via apoptosis or differentiation), can be calculated as 2^average division number^. The actual increase in the number of IL-13^+ ^T cells from control subjects was 87 ± 14% lower (*n *= 6) than the theoretical maximum increase; the actual increase in IL-13^+ ^T cells from asthmatics was 43 ± 35% lower (*n *= 6) than the theoretical maximum, significantly lower than that for control subjects (*p *= 0.03). These data indicate that during culture there are losses of the progenitors of IL-13^+ ^T cells, and that this loss of progenitors is greater in female control subjects than in female asthmatics (Figure 5B). Indeed, in cultures derived from female asthmatics, few IL-13^+ ^T cell progenitors are lost--approximately one-quarter the loss of progenitors that occurs for control subjects. There were no significant differences between male asthmatics and male controls (and male and female controls overall) for any of these CFSE-derived parameters (not shown). Therefore, reduced loss of IL-13^+ ^T cells rather than increased proliferation is responsible for increased accumulation of IL-13^+ ^T cells in PBL from female asthmatics.

IFN-γ^+ ^T cells from asthmatics did not proliferate more than those from control subjects (Figure 5B). The majority (~80%) of IFN-γ^+ ^T cell progenitors from control subjects were lost during culture of PBL from control subjects. As with IL-13^+ ^T cells, the loss of IFN-γ^+ ^T cell progenitors is significantly reduced in asthmatic subjects. Therefore, the increased accumulation of the IFN-γ^+ ^T cell subset from asthmatics, relative to control subjects, is a function of increased maintenance of T cell subset progenitors rather than enhanced proliferation.

## Discussion

Our study provides the first evidence to date that T cells from atopic asthmatic and nonasthmatic control subjects differ in their accumulation of T cell subsets in response to CD3+CD28-mediated and antigen-independent stimulation. Moreover, gender plays a significant role in determining these responses in type 2 T cell accumulation.

IL-2 stimulated increases in IL-13^+ ^T cells were significantly higher in PBL from asthmatics than in PBL from control subjects, and this increase is almost totally attributable to the female population of asthmatics studied. That the increased accumulation of IL-13^+ ^T cells was observed in female but not male asthmatics, and the association was maintained when analysis was restricted to only atopic subjects, indicates that the effect was a function of asthma and not atopy per se. Whether there is also an effect of atopy per se on accumulation of IL-13+ T cells should be tested next, by comparing nonatopic nonasthmatic and atopic nonasthmatic healthy control subjects. CD3+CD28-stimulated accumulation of IL-13^+ ^T cells was also greater in female subjects, but was not influenced by asthma status. IFN-γ^+ ^T cell accumulation, when stimulated by either CD3+CD28 or IL-2, was significantly greater in cells from asthmatics relative to controls, with no effect of gender. Interestingly, in "Th1-polarizing" conditions including IL-12, IFN-γ+ T cells increased to similar extents in both asthma and control groups. We would hypothesize that the impact of IL-12 is partially redundant. In cultures with IL-12, existing IFN+ cells survive to a greater extent than in the absence of IL-12 for controls, but in asthmatics survival is already near its maximal limit so as to not be further increased by IL-12.

Unlike the majority of T cells (IL-13^- ^and IFN-γ^+ ^subsets), IL-13^+ ^T cells increase in number and proportion when stimulated with IL-2, even in the absence of antigenic stimulation. This accumulation occurs via selective proliferation of the IL-13^+ ^T cell subset [12,15]. In female asthmatic subjects, IL-13^+ ^T cells accumulate to even greater numbers, with about a 4-fold greater increase than that observed in female control and male asthmatic subjects. CFSE analyses demonstrate the mechanism is not by a further increase in IL-2-stimulated proliferation, but rather via a reduction in the loss of IL-13^+ ^T cells during culture.

Our data demonstrating enhanced accumulation of not only IL-13^+ ^but also IFN-γ^+ ^T cells from asthmatic subjects in response to IL-2 suggest the importance of antigen-independent stimulation of T cells in asthma. Cytokine-stimulated (bystander) accumulation of T cells may potentially explain allergen-independent sensitization in asthmatics, e.g., respiratory infection leading to an IL-2- or IL-15-induced proliferation of resident memory, viral antigen-non-specific, allergen-specific type 2 T cells, conditions which have been speculated to be relevant to the development of asthma [19]. Viral infections have been implicated in asthma pathogenesis and exacerbations by numerous epidemiologic studies, although limited mechanistic insight exists [1,3,4,7,8]. Pathogens (bacteria, virus) induce cytokine production by monocytes, macrophages, and dendritic cells (i.e., monocytic cells). Tissue damage (e.g., products of necrotic cells; endothelins released by endothelia or epithelia, platelet-derived factors), independent of pathogen infection, can also induce cytokine production by monocytic cells [20,21]. IL-15, a cytokine produced by monocytic cells, is capable of inducing antigen-independent, bystander proliferation of IL-13^+ ^T cells (unpublished observations). These bystander events need not necessarily take place in the airways. They may also occur in the lymph nodes that drain the lungs where both previously activated memory cells and naïve, type 2 T cells could either be increased in number prior to being stimulated by allergen or trafficking to the lungs. Thus, although starting numbers of IL-13^+ ^T cells did not significantly differ between asthmatics and non-asthmatics, enhanced bystander accumulation of type 2 T cells may nevertheless lead to an acute increase in numbers of IL-13^+ ^T cells in asthmatics during allergen- or pathogen- induced immune responses.

Increased accumulation of both IL-13^+ ^and IFN-γ^+ ^in asthmatics contradicts the Th2 hypothesis of asthma, yet is consistent with observations of IFN-γ being elevated (with type 2 cytokines) in some asthmatics [4,5]. It has been suggested that microbial infections can contribute to the development of allergic asthma, as well as to exacerbations in subjects with established asthma [1,3,4,7,8]. In asthmatic compared to nonasthmatic subjects, microbe-specific IFN-γ^+ ^T cells can increase to greater numbers during infection. Additionally, non-microbe specific IFN-γ^+ ^T cells could also increase in asthmatics, potentially increasing any allergen-specific IFN-γ^+ ^T cells as well. An acute increase in IFN-γ production during an allergic reaction, or reaction to microbial infection, could exacerbate inflammation in asthmatics by increasing the recruitment and activation of immune cells [6].

The mechanism contributing to gender differences in IL-13^+ ^T cell accumulation is unclear. Between post-puberty and pre-menopause years, females have a higher incidence of developing asthma than males (reviewed by Balzano et al. [18]). However, during pre-puberty, asthma development tends to occur somewhat more frequently in boys. A portion of adult, asthmatic women also experience perimenstrual worsening of asthma symptoms [22]. These observations suggest the involvement of female sex hormones in the increased accumulation of IL-13^+ ^T cells. Potential explanations for enhanced IL-13^+ ^T cell accumulation selectively in female asthmatics is an interaction between high levels of female sex hormones and the asthma/atopic environment or asthma/atopy susceptibility genetic polymorphisms. The effects of gender and asthma on IL-13^+ ^T cells could be either from epigenetic, developmental imprinting from continuous hormone exposure, or, alternatively, from acute stimulation by hormones before blood draw. Interestingly, increased accumulation of IL-13^+ ^T cells under CD3+CD28-stimulatory conditions was also observed in females, but was in this case independent of asthma status. It is tempting to speculate if hormonal-driven epigenetic changes to IL-13^+ ^T cells in females may have broader consequences for susceptibility to type 2 T cell-associated autoimmune diseases that predominantly occur in females, e.g., lupus and multiple sclerosis.

Decreased numbers of regulatory T cells have been hypothesized to contribute to the development of asthma [11]. Corticosteroid use is suggested to decrease the development of adaptive IL-10-producing T_R_1 regulatory T cells [11]. Perhaps female sex hormones may also contribute to decrease T_R_1 development and subsequent hyperresponsiveness of IL-13^+ ^T cells in female asthmatics. The main mechanism described for inhibition of T cell accumulation by regulatory T cells is inhibition of proliferation. Increased accumulation of IL-13^+ ^and IFN-γ^+ ^T cells in asthmatics resulted from decreased loss of cytokine^+ ^cells rather than increased proliferation relative to controls. Also, increased accumulation of IL-13^+ ^T cells in female asthmatics was observed only for the IL-2-stimulated condition and not the CD3+CD28-mediated condition. For these reasons it seems less likely that differences in regulatory T cells account for the observed increased accumulation of T cell subsets in asthmatics.

## Conclusions

In conclusion, our data demonstrate that both type 1 and type 2 T cell subsets increase to a greater extent in asthmatic than control subjects in response to antigen-independent, cytokine-mediated stimulation, with the increased accumulation of type 2 T cells exclusively in female asthmatics. These results provide explanations for the higher frequency of adult asthma in females and for the apparently paradoxical increase of both IFN-γ and IL-13 in the lungs of a subset of allergen-challenged asthmatics.

## Abbreviations

ANCOVA: analysis of covariance; CFSE: carboxyfluorescein diactetate succinimydl ester; FEV_1: _forced expiratory volume at 1-sec; FVC: forced vital capacity; *GLM: *General Linear Model; IFN: interferon; IL: interleukin; IQR: interquartile range; PBL: peripheral blood lymphocytes; PC_20_: provocative concentration of methacholine causing 20% fall in FEV_1_; PMA: phorbol myristate acetate; PP: percent predicted

## Competing interests

The authors declare that they have no competing interests. All contributions for this manuscript by MJL were completed at Wake Forest University Health Sciences. MJL is currently employed by Centocor R&D.

## Authors' contributions

MJL and RBP wrote the manuscript. MJL and SF performed all experiments. SF, SPP. SF, ERB, and SPP recruited and characterized clinical status of subjects. All authors contributed to experimental design, data interpretation and formation of the manuscript in its final form.

## Supplementary Material

Additional file 1 **methods, table S1, figure S1, additional file references**. Click here for file
