# Supplementary material for: Asthma and gender impact accumulation of T cell subtypes
Source: Respir Res. 2010 Jul 28;11(1):103. doi: 10.1186/1465-9921-11-103 (PMC2919468; doi:10.1186/1465-9921-11-103)
Supplement: Additional file 1 — methods, table S1, figure S1, additional file references. [file 1465-9921-11-103-S1.DOC]

**ADDITIONAL FILE 1**

**(methods, TABLE s1, FIGURE s1, AddiTIONAL FILE REferences)**

**Methods**

**Qualification criteria for atopic asthma population**

To qualify as atopic asthmatic, subjects must have met the following criteria: 1) bronchial hyperreactivity, defined as a methacholine PC_20_ (provocative concentration causing 20% fall in forced expiratory volume at 1-sec (FEV_1_)) of <8 mg/ml, or reversibility of airway obstruction, defined as an inhaled albuterol-induced >12% increase in FEV_1_; 2) symptoms consistent with asthma (e.g., cough, wheeze, shortness of breath); 3) physician’s diagnosis of asthma without a diagnoses of any other pulmonary disorder; 4) atopy determined by serum IgE measurements (>50 IU/mL) and skin prick tests to a panel allergens (>3mm wheal for at least one allergen); 5) nonsmoker (>12 months non-smoking) and less than 3 pack-years cigarette exposure (pack-years = years smoking x average number of packs smoked daily). Measurements and interpretations of these criteria are as reported ^1;2^.

**Details for culture conditions**

Peripheral blood lymphocytes (PBL) were isolated from peripheral venous blood by Ficoll-Histopaque density gradient centrifugation and non-adherences to plastics, as previously reported ^3^. For IL-2- and CD3+CD28- stimulated cultures, 2 x 10^6^ and 0.5 x 10^6^ PBL were used, respectively. For CD3+CD28-stimulated cultures, CD3 monoclonal antibody was bound to wells by incubating non-tissue culture-treated plates with 1 μg/ml clone OKT3 in 50mM TRIS, pH 9.5 at 4°C for 18-h. Wells were then washed with phosphate buffered saline and blocked 10% fetal bovine serum for 30min before addition of cells. CD28 monoclonal antibody (clone 9.3) was provided at 2 μg/ml in solution. After 2-d of culture in CD3-coated wells, cells were transferred to wells of a tissue culture-treated 24 well plate. Because of variability among subjects in proportions of T cells capable of IL-2 production upon CD3+CD28-mediated stimulation, IL-2 was added to the CD3+CD28-stimulated cultures to eliminate a confounding influence on the proliferation of T cells.

IL-12-neutralizing monoclonal antibody was included in all conditions (except those with added IL-12) to eliminate confounding effects of variable levels of IL-12 produced by the few, if any, contaminating monocytes.

**Calculation of average division numbers and minimum progenitor numbers**

After culture of carboxyfluorescein diactetate succinimydl ester (CFSE)-labeled cells, IL-13, IFN-γ and CD3/CD5 were detected with PE-, APC-, and TriColor- labeled monoclonal antibody, respectively. As cells divide, the intensity of CFSE fluorescence halves, with each halving indicating a discrete division number. From analysis of numbers of cytokine^+^ T cell in each division region, average division numbers and minimum progenitor numbers can be calculated ^4;5^. Progenitors are the cells present at the start of culture from which the cells detected at the end of culture derive. Briefly, the average number of divisions that the progenitors of the population of interest undergo is calculated as: **D = [Σ(CFSE_n_ cell % * n)]/100**, where n = division number based on discrete CFSE peaks, and CFSE cell % = the percentage of cells in the population of interest that are in the CFSE peak corresponding to n divisions. Minimum “progenitor number” is calculated using the formula: **P= Σ(CFSE_n_ cell # /2^n^),** where n=division number based on discrete CFSE peaks, and represents the minimal number of cells necessary to produce the total number of CFSE-labeled cells. The difference between progenitor number and the number of cytokine^+^ cells at day 0 represents the “loss of progenitors”.

**Table S1.** Demographic and spirometry data for asthmatic subjects.

| Parameter | Statistic | Asthmatics | Male Asthmatics | Female Asthmatics | p-value ^*^ |
| --- | --- | --- | --- | --- | --- |
| Subjects | *n* | 25 | 12 | 13 |  |
| Race, *n* | Cauc. / Afr.Am. ^†^ | 15 / 9 ^C^ | 8 / 4 | 7 / 5 ^‡^ |  |
| Age | Mean±SD (range) | 34±9 (18–51) | 33±9 (20-50) | 35±10 (18–51) | 0.52 |
| PC20 | Geo.Mean (range) | 1.6 (0.1–8.0) | 1.7 (0.2–7.2) | 1.4 (0.01–8.0) | 0.66 |
| FEV_1_  % predicted | Mean±SD (range) | 76±20 (26–110) | 72±21 (26–102) | 80±20 (31–110) | 0.29 |
| FVC  % predicted | Mean±SD (range) | 87±18 (46–109) | 87±21 (46–108) | 88±16 (5 –109) | 0.86 |
| FEV_1_/FVC | Mean±SD (range) | 0.7±0.1 (0.45–0.87) | 0.67±0.09 (0.45–0.77) | 0.73±0.11 (0.48–0.87) | 0.15 |
| % FEV_1_ Reversibility | Median (range) | 16 (0–100) | 16 (7–54) | 14 (0 – 100) | 0.71 |

^*^ p-values for differences between male and female asthmatics were determined by t-tests for means and geometric means, and Mann-Whitney tests for medians. In addition to listed parameters, there were no significant differences (p>0.1) among male and female asthmatics for: IgE levels (geometric mean), sputum eosinophil number, body mass index, day 0 % IL-13^+^ T cells, and day 0 % IFN-γ^+^ T cells. Differences in eosinophil numbers did show a trend (p=0.088).

^†^ Abbreviations: FEV_1_, forced expiratory volume at 1 second; PC20, provocative concentration of methacholine inducing a 20% decrease in FEV_1_; FVC, forced vital capacity; FEV_1_ reversal, % increase in FEV_1_ above baseline induced by inhaled albuterol.

^‡^ One female asthmatic was of mixed race.


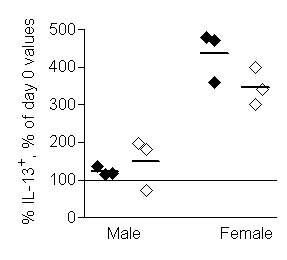


CD8^−^

CD8^+^

**Figure S1. Accumulation of IL-13^+^ cells in CD4^+^ and CD8^+^ T cell subsets from asthmatics.** Data obtained in the experiments described in Figure 3 for asthma subjects, for which CD8 staining was available, were analyzed for accumulation of IL-13**^+^** T cells in CD8**^-^** (filled symbols) and CD8**^+^** (open symbols) T cell subsets. Each symbol indicates the proportion of IL-13**^+^** cells, expressed as a percent of the respective day 0 values for each subject after 6-d culture with IL-2 + anti-IL-12.

**Additional File References**

1. Deykin, A., M. E. Wechsler, H. A. Boushey, V. M. Chinchilli, S. J. Kunselman, T. J. Craig, E. Dimango, J. V. Fahy, M. Kraft, F. Leone, S. C. Lazarus, R. F. Lemanske, R. J. Martin, G. R. Pesola, S. P. Peters, C. A. Sorkness, S. J. Szefler, and E. Israel. 2006. Combination Therapy with a Long-acting {beta}-Agonist and a Leukotriene Antagonist in Moderate Asthma. *Am.J Respir.Crit Care Med.* In press. doi:10.1164/rccm.200601-112OC.

2. Lester, L. A., S. S. Rich, M. N. Blumenthal, A. Togias, S. Murphy, F. Malveaux, M. E. Miller, G. M. Dunston, J. Solway, R. L. Wolf, J. M. Samet, D. G. Marsh, D. A. Meyers, C. Ober, and E. R. Bleecker. 2001. Ethnic differences in asthma and associated phenotypes: collaborative study on the genetics of asthma. *J Allergy Clin Immunol* 108:357-362.

3. Perussia, B. and M. J. Loza. 2005. Purification of peripheral blood natural killer cells. *Methods Mol.Med.* 107:147-161.

4. Loza, M. J. and B. Perussia. 2001. Final steps of natural killer cell maturation: a model for type 1-type 2 differentiation? *Nat.Immunol.* 2:917-924.

5. Loza, M. J. and B. Perussia. 2003. Accumulation of type 2 cytokine+ T cells: differentiation-independent proliferation of pre-existing type 2 T cells. *Eur.J.Immunol.* 33:939-949.
